# Comparison of Dexmedetomidine and Ketamine in Serratus Anterior Plane Block for Postoperative Pain Control in Thoracotomy Patients: A Randomized Clinical Trial

**DOI:** 10.5812/aapm-137664

**Published:** 2024-02-17

**Authors:** Mahbobeh Rashidi, Kamran Mahmoodi, Reza Akhondzadeh, Reza Baghbanian, Fatemeh Jahangiri Mehr, Niloofar Safaei Semnani

**Affiliations:** 1 Department of Anesthesiology and Pain Medicine, Pain Research Center, Faculty of Medicine, Ahvaz Jundishapur University of Medical Sciences, Ahvaz, Iran; 2 Department of Epidemiology and Biostatistics, School of Public Health, Isfahan University of Medical Sciences, Isfahan, Iran

**Keywords:** Serratus Anterior Plane Block, Dexmedetomidine, Ketamine, Thoracotomy, Postoperative Pain

## Abstract

**Background:**

Postoperative pain control after thoracotomy is very important, and if not controlled, it can cause severe complications.

**Objectives:**

This study aimed to compare dexmedetomidine and ketamine in serratus anterior plane block (SAPB) in pain control after thoracotomy.

**Methods:**

This randomized clinical trial was conducted on 74 patients aged 18 to 60 years old with American Society of Anesthesiologists (ASA) class I or II who were referred to Imam Khomeini hospital in Ahvaz, Iran, for thoracotomy and randomly divided into two groups. After surgery, the SAPB with ultrasound-guided was performed. In the ropivacaine-ketamine (RK) group, ketamine 0.5 mg/kg and 0.4 cc/kg ropivacaine solution 0.25% and in the ropivacaine-dexmedetomidine (RD) group, in addition to 0.4 cc/kg ropivacaine 0.25%, dexmedetomidine 0.5 µg/kg was added. Verbal Numeric Scale (VNS), systolic blood pressure (SBP), diastolic blood pressure (DBP), respiratory rate (RR), heart rate (HR), and mean arterial blood pressure (MAP) were recorded.

**Results:**

There was no significant difference in both groups in terms of demographic information (P < 0.05). The average VNS was lower in the ketamine group than in the dexmedetomidine group; however, there was a significant difference only at 1, 12, and 24 hours after surgery (P < 0.05). There was no statistically significant difference between the two groups in terms of SBP and DBP, HR, and MAP. There was a significant difference in the RR in the two groups at 12 and 24 hours after the operation (P < 0.05).

**Conclusions:**

Dexmedetomidine and ketamine, which were used as supplements to ropivacaine for SAPB in patients undergoing elective thoracotomy, reduced the pain intensity after thoracotomy; nevertheless, the intensity of pain reduction was more and more effective in the group receiving ketamine.

## 1. Background

Postoperative pain, or pain after surgery, especially chest surgery, is a natural physiological response to the surgical intervention. It prolongs the healing process and discharge of the patient, resulting in wound infection and cardiovascular and respiratory problems (1, 2). Acute postoperative pain is secondary to inflammation caused by trauma to the tissue or direct damage to the nerve. It can be divided into two groups: Nociceptive and neuropathic pain. Nociceptive pain can have a somatic cause (e.g., localized pain caused by muscles, bones, skin, joints, and connective tissue) or a visceral cause (e.g., dull pain caused by hollow organs). Neuropathic pain, on the other hand, can be due to damage to the central or peripheral nervous system (e.g., polyneuropathies and mononeuropathies) (3). 

There are a variety of methods for acute postoperative pain control after thoracotomy. These methods include systemic opioids, intercostal nerve block (ICB), novel fascial plane block procedure, including serratus anterior plane block (SAPB), pectoral nerve block, and erector spinae plane block. 

There is compelling evidence that the use of SAPB and fascial plane blocks is superior to the use of systemic opioid-based regimens (4). Traditionally, opioids have been used to control pain. However, with a better understanding of the mechanisms of pain perception in newer methods, several mechanisms can be used simultaneously. For example, topical pain relievers directly block the activity of pain receptors. Anti-inflammatory drugs, such as acetaminophen, pregabalin, gabapentin, dexmedetomidine, and clonidine, are used to inhibit pain by targeting specific neurotransmitters (3). The duration of the effect of a peripheral nerve block (PNB) is limited. The use of non-opioid auxiliary drugs, such as dexamethasone, alpha-2 agonists, non-steroidal anti-inflammatory drugs, and midazolam, can prolong the duration of the local anesthetic block (5). 

Ketamine is an N-methyl-D-aspartate (NMDA) antagonist and has strong analgesic effects (6), which can be attributed to its effect on a number of opioid, muscarinic, and NMDA receptors (7). Administering ketamine with ropivacaine or levobupivacaine increases the intensity and duration of spinal anesthesia (6). Dexmedetomidine is an alpha-2 receptor agonist whose analgesic effect seems to be related to the release of acetylcholine. These drugs have a strong analgesic effect, and their use, together with analgesics, can even increase their effectiveness. Additionally, alpha-2 agonists reduce the adverse effects of opioid analgesics, such as physiological and psychological effects caused by drug withdrawal (8-10). Ropivacaine is a long-acting topical pain reliever with lipid solubility that can block nerve fibers of pain (δ A and C fibers) and inhibit various receptors, enhance the release of glutamate, and depress the activity of certain intracellular signaling pathways (11). The present study evaluated two groups of patients who underwent thoracotomy and were transferred to the intensive care unit (ICU) after surgery and received ultrasound-guidance SAPB using a combination of either ketamine-ropivacaine or dexmedetomidine-ropivacaine and the effect of these two combinations in controlling postoperative pain.

## 2. Methods

This randomized clinical trial was carried out on 74 patients aged 18 to 60 years old with American Society of Anesthesiologists (ASA) class I or II who were referred to Imam Khomeini hospital in Ahvaz, Iran, within 2022/12/11 and 2023/03/20. The protocol of this study was reviewed and approved by the Ethics Committee of Ahvaz Jundishapur University of Medical Sciences, Ahvaz, Iran, and then registered on the Iranian Registry of Clinical Trials (IRCT20190417043295N3). After obtaining the study ethics code and registering the study as a randomized clinical trial (RCT), all patients referred to the Imam Khomeini Medical Training Center of Ahvaz and underwent thoracotomy surgery were evaluated in terms of the conditions of entering and exiting the study. The inclusion criteria included the physical condition with ASA class 1 and 2 and an age range of 18 to 60 years. The exclusion criteria included the infection at the injection site, allergy to local anesthetic, body mass index (BMI) above 40, drug, alcohol, or substance abuse, pregnancy or breastfeeding, coagulopathies, history of heart failure (HF), heart conduction disorders (except 1st-degree block) and atrial fibrillation (AF) suffering from hepatorenal diseases, and a history of neuropsychological diseases. 

First, the study protocol was explained to the patients; then, informed consent was obtained, and the patients were randomized into two groups by block randomization. Thoracotomy operations are usually performed within several months of sampling, and the required 74 patients will not undergo such an operation in the hospital at the same time. A total of 74 sealed envelopes with cards inside that include 37 cards number 1 and 37 cards number 2 are prepared, and each patient is randomly selected for the group in which they will be studied. 

Description of groups: (1) the prescription of ropivacaine and dexmedetomidine; (2) the prescription of ropivacaine and ketamine. 

**Figure 1.**
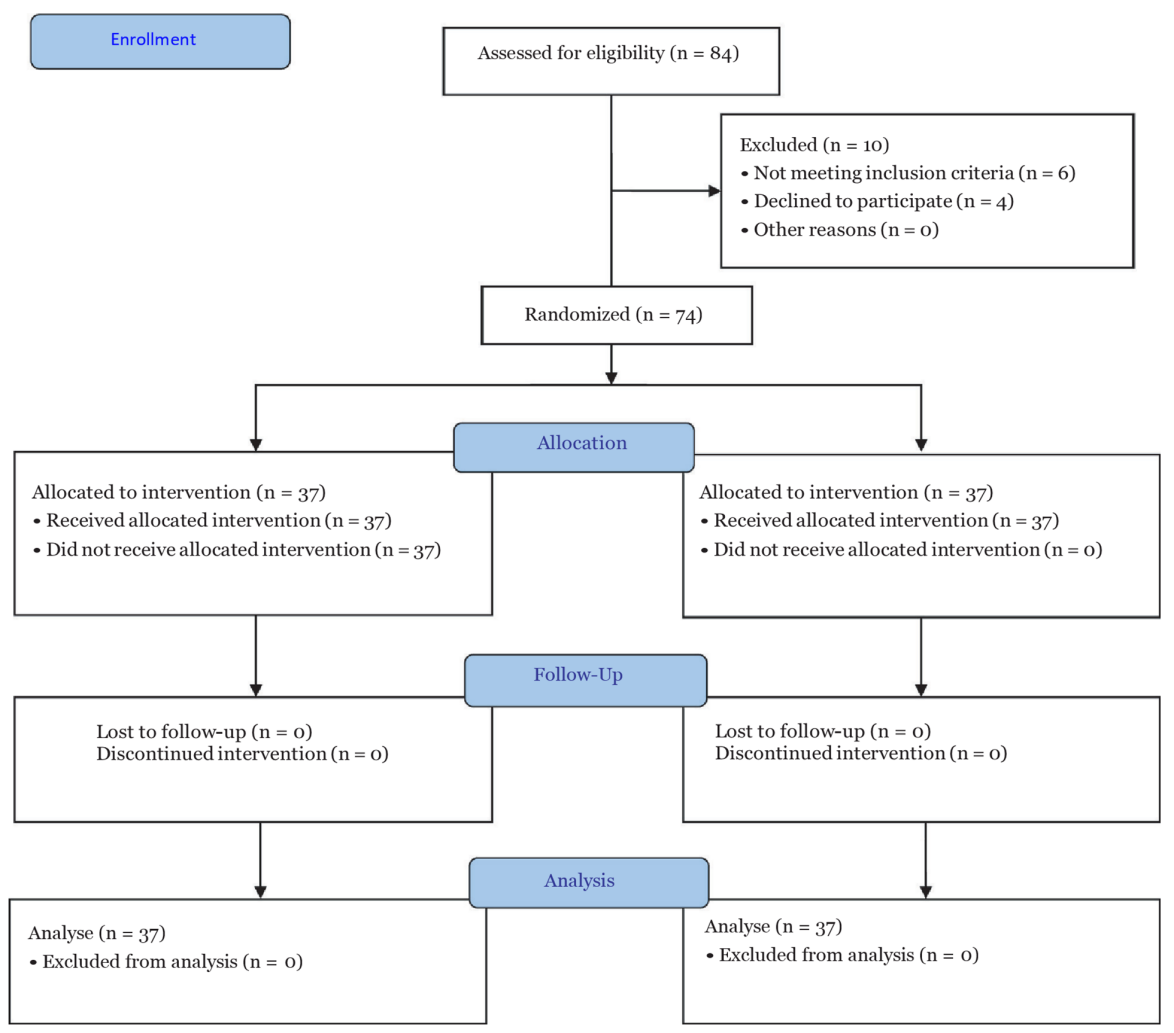
CONSORT flow diagram

The patients and evaluators were unaware of the type of intervention. According to the information obtained from the study by Agamohammdi et al. (12) and using the formula for determining the sample size for two-group studies, a random sample of 37 per group and 74 in total was determined (Figure 1). 


$$37≅n=\frac{{\left(Z_{1-\frac{\alpha }{2}}+Z_{1-\beta }\right)}^{2}.\left(S_{1}^{2}+S_{2}^{2}\right)}{{\left(\mu_{1}-\mu_{2}\right)}^{2}}\;$$

S_1_ = 2.45, S_2_ = 3.54, μ_1_ = 4.9, μ_2_ = 2.89, α = 0.05, β = 0.2.

### 2.1. Anesthesia Protocol 

All patients were nil per os for at least 8 hours before thoracotomy. After coming to the operating room (OR), the patients were evaluated by non-invasive blood pressure (NIBP), heart rate (HR), blood oxygen saturation (SpO_2_), and electrocardiogram (ECG). These drugs were used for the induction of general anesthesia: Midazolam (0.03 mg/kg), fentanyl (2 µg/kg), propofol (2 mg/kg), and then cis-atracurium (0.15 mg/kg). After intubation, isoflurane (1 MAC) was prescribed to the patients intraoperatively. Then, end-tidal carbon dioxide (ET CO_2_) was maintained at 35 - 45 mmHg during surgery. The tidal volume was placed on 7 - 10 mL/kg. Remifentanil (0.1 µg/kg/min) was infused during surgery. When the operation was completed and the patients were extubated, they were transferred to the post-anesthesia care unit (PACU). 

### 2.2. Ultrasound-Guided Serratus Anterior Plane Block 

In PACU, after hemodynamic stability, the patient is positioned supine or lateral decubitus. A linear high-frequency ultrasound transducer is placed in the sagittal or transverse position at the midaxillary line. Under continuous ultrasound imaging, the ultrasound transducer moved posteriorly, and latissimus dorsi, serratus anterior muscles, and the fascial plane were identified between them and the ribs and pleura.  

**Figure 2.**
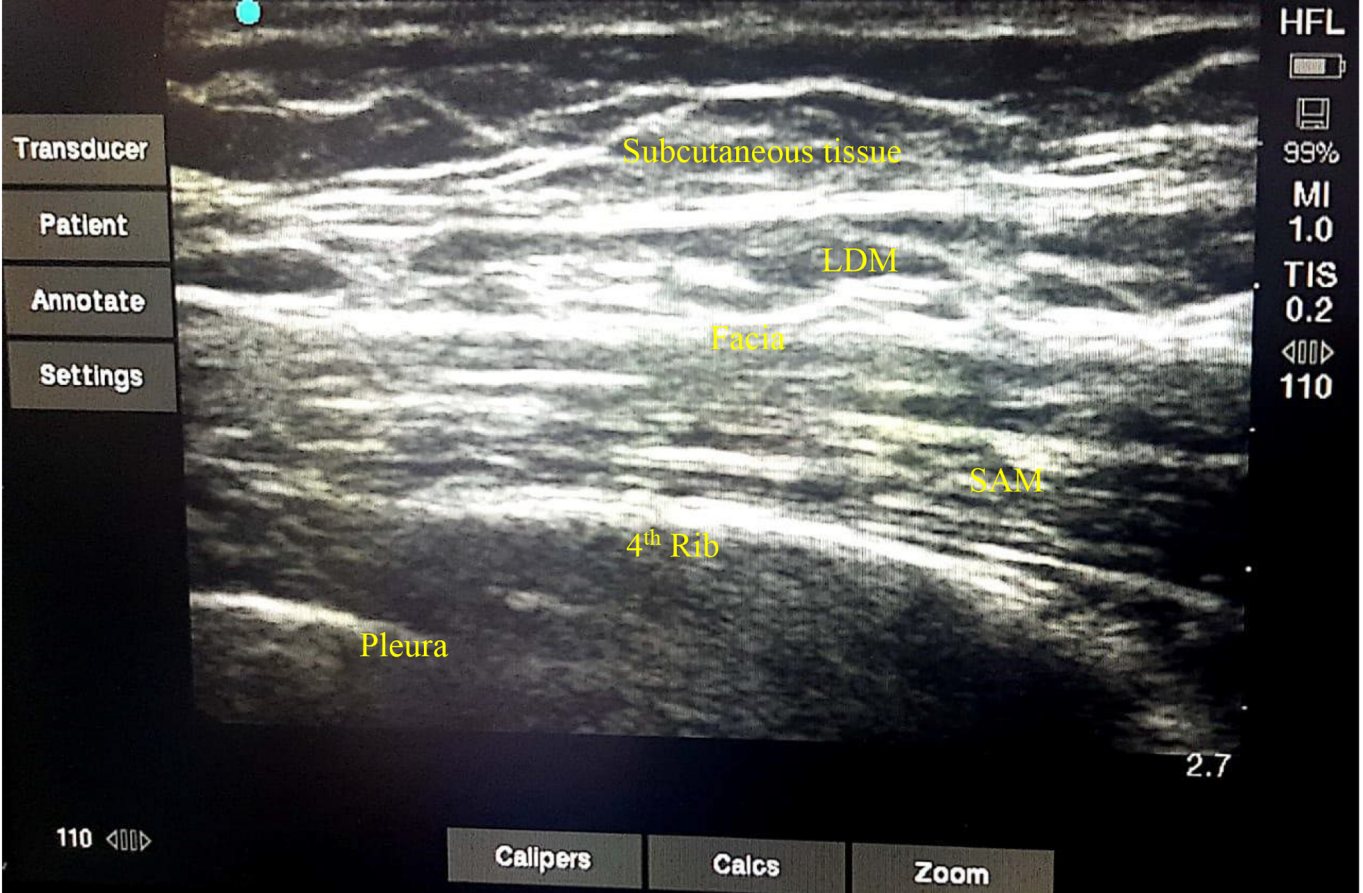
Ultrasound view of serratus anterior plane block at the mid-axillary line. SAM, serratus anterior muscle; LDM, latissimus dorsi muscle.

The skin was prepped and draped, and a 3.5-inch (22-gauge) needle was advanced using an in-plane or out-of-plane approach. After careful aspiration, half of the solution was injected superficially into the serratus anterior muscle (deep to the latissimus dorsi muscle), and another half was injected deep into the serratus anterior. Then, the needle was removed, and a wound pressure dressing with an ice pack was placed at the site of injection (Figure 2). 

The pain fellowship was aware of the injected drug in each group but had no role in the data collection and analysis. After transfer to the ICU, study measures were recorded. 

### 2.3. Measures 

The primary objective was pain score (verbal numeric scale [VNS]), and secondary objectives were systolic blood pressure (SBP), diastolic blood pressure (DBP), mean arterial blood pressure (MAP), respiratory rate (RR), and HR. Measured variables were recorded five times: Before intervention (time 0), 1st hour, 6th hour, 12th hour, and 24th hour after the intervention. The pain score was ranked via VNS. This scale is rated from 0 to 10, indicating no pain and 10 the worst pain imaginable. When the VNS score was higher than 3, the patient received 1 mg/kg meperidine. 

### 2.4. Statistical Analysis 

The sample size was calculated as 37 patients per group were required. Statistical analysis was performed using GraphPad Prism (version 8) and SPSS (version 18.0, IBM). The analysis outputs were defined as mean ± standard deviation (SD). The comparisons within groups and between groups (at different times) were performed using the repeated measures of analysis of variance (ANOVA), and between a number of variables, the Chi-square test was performed. The significance level of all tests was determined with a p-value of less than 0.05.

## 3. Results

In this study, of 74 patients, 46 (62.2%) and 28 (37.8%) subjects were male and female, respectively, with a mean age of 40.14 ± 11.897 (range: 19 to 65 years). The patients’ demographic characteristics are shown in Table 1. No significant differences were observed in demographic characteristics (age, gender, weight, height, and BMI) (P > 0.05).

**Table 1.** Patients’ Demographic Characteristics ^a^

Characters	Groups		P-Value
	RK	RD	
**Gender (M/F) **	11.26	17.20	0.231
**Age **	38.43 ± 11.261	41.84 ± 12.420	0.976
**Height (cm) **	171.729 ± 8.338	173.459 ± 8.886	0.651
**Weight (kg) **	77.02 ± 17.34	84.24 ± 13.43	0.212
**BMI (kg/m** ^**2**^ **) **	26.05 ± 4.090	27.94 ± 3.25	0.812
**Operation time (h) **	2.5 ± 0.96	2.7 ± 1.1	0.272

Abbreviations: RK, ropivacaine-ketamine; RD, ropivacaine-dexmedetomidine; BMI, body mass index.

^a^ Values are expressed as mean ± SD unless otherwise indicated.

Table 2 compares the average score of VNS between the two groups. At baseline and before the block, the RD group had more pain than the RK group; however, this difference was not significant (P = 0.793). Compared to baseline scores, there was a significant decrease in VNS scores in the two groups during measurement times. The statistical VNS score was higher in the RD group at most times except in the 6th hour. However, at the 1st, 12th, and 24th hours, the VNS score had significant differences between the two groups (P < 0.05). 

**Table 2.** The Mean Verbal Numeral Rating Scale ^a^

Time	Groups		P-Value
	RK	RD	
**Pre-block **	8.59 ± 1.34	8.30 ± 1.35	0.793
**1st hour **	1.92 ± 1.42	1.14 ± 0.347	< 0.001
**6th hour **	1.19 ± 0.616	1.35 ± 0.588	0.116
**12th hour **	1.05 ± 0.329	2.27 ± 1.122	< 0.001
**24th hour **	1.03 ± 0.164	4.95 ± 0.998	< 0.001

Abbreviations: VNS, Verbal Numeric Scale; RK, ropivacaine-ketamine; RD, ropivacaine-dexmedetomidine.

^a^ In the comparison of the two receiving RD and RK groups, there is no significant difference between them in MAP except for the 12th hour.

Table 3 shows the comparison between these values. At all times, MAP was lower in the RD group except for the time of the 24th hour.

**Table 3.** The Mean Arterial Blood Pressure

Time	Groups		P-Value
	RK	RD	
**Pre-block **	97.35 ± 6.83	97.34 ± 6.91	0.758
**1st hour **	95.66 ± 8.92	93.73 ± 8.58	0.204
**6th hour **	87.37 ± 6.51	86.92 ± 8.88	0.343
**12th hour **	89.55 ± 8.26	87.81 ± 6.85	0.029
**24th hour **	87.58 ± 8.48	93.10 ± 7.60	0.252

Abbreviations: RK, ropivacaine-ketamine; RD, ropivacaine-dexmedetomidine.

Table 4 compares HR between the two study groups. The heart rate was higher in the RK group at most times.

**Table 4.** The Mean Heart Rate

Time	Groups		P-Value
	RK	RD	
**Pre-block **	102.73 ± 12.09	91.95 ± 9.48	0.010
**1st hour **	92.92 ± 10.82	84.46 ± 9.48	0.326
**6th hour **	87.81 ± 8.65	75.84 ± 10.69	0.477
**12th hour **	84.43 ± 7.69	76.57 ± 8.27	0.795
**24th hour **	90.97 ± 7.13	82.51 ± 7.56	0.813

Abbreviations: RK, ropivacaine-ketamine; RD, ropivacaine-dexmedetomidine.

## 4. Discussion

Postoperative pain management following thoracotomy is of paramount importance, as uncontrolled pain might lead to severe complications. This study aimed to compare the efficacy of dexmedetomidine versus ketamine in SAPB for pain control after thoracotomy surgery. The results indicated that SAPB was highly effective in both groups, notably reducing pain within the first hour after the intervention. This pain reduction persisted for up to 24 hours in patients receiving ketamine-ropivacaine. The addition of ketamine to ropivacaine (RK group) increased analgesia and reduced pain in this group, compared to the RD group, within the initial 24 hours. The RK group reported less pain at the 6th, 12th, and 24th hours post-drug administration.

Talebi et al.'s study (13) on the transverse abdominal plane (TAP) block revealed that a combination of marcaine (20 cc of 0.125% solution) and dexmedetomidine (0.5 μg/kg) reduced pain intensity and analgesic use in the first 24 hours after surgery. Mahmoudi et al.'s investigation of intercostal block with added dexmedetomidine (0.5 μg/kg) to ropivacaine (5 cc of 0.25% solution) in thoracotomy patients demonstrated a reduction in opioid use within the first 48 hours (14). Omar Mostafa et al., in the paravertebral block for postoperative analgesia in mastectomy, utilized 1 μg/kg dexmedetomidine with bupivacaine, reporting a reduction in postoperative pain intensity (15). A study by Habibi et al. in Iran examined the effect of 1 μg/kg dexmedetomidine as a bolus and 0.5 - 1 μg/kg/hour as an infusion on acute pain after cardiothoracic surgeries, revealing significantly lower pain scores in the dexmedetomidine group in the 24 hours after surgery (16).

In line with the results of the present study, Margulis et al., in ultrasound-guided arthroscopic shoulder surgery, compared the addition of dexamethasone and dexmedetomidine to ropivacaine, reporting a decrease in analgesic consumption in the first 48 hours, similar to the results of the current study (17). Another study in Iran conducted by Agamohammdi et al. on 64 patients compared the effect of bupivacaine to the combination of bupivacaine with dexmedetomidine, showing greater and faster pain reduction in the combination group (12). Similarly, Akhondzadeh et al., in a supraclavicular block, used 1 μg/kg dexmedetomidine with lidocaine, prolonging analgesia and decreasing the total analgesic dose, compared to lidocaine alone (18).

In accordance with the results of the present study, Borys et al. compared the administration of intravenous ketamine (1 mg/kg) before surgical incision to a paravertebral block using 30 cc bupivacaine 0.25%, finding significantly less acute pain intensity and reduced morphine consumption in the paravertebral block group. Moreover, patients were more satisfied with the postoperative management of paravertebral block, and the intensity of post-thoracotomy pain syndrome (PTPS) decreased during a 6-month postoperative period (19).

However, in a cohort study in China, Pangthipampai et al. compared the paravertebral block of the chest with and without the administration of intravenous ketamine in mastectomy surgery. Although effective in only 20% of patients, the combination of 4 cc ropivacaine 0.5% and dexmedetomidine (0.1 - 0.5 μg/kg/hour) was successful in those who experienced pain during the separation of breast tissue from the pectoralis major muscle, requiring a supplemental dose of intravenous ketamine (20.5 ± 38 mg) (20).

Based on the results of Yazigi et al., no reduction in acute pain score and supplemental morphine consumption following thoracotomy was observed in patients receiving the combination of ketamine and intercostal block (21). In contrast to the results of the present study, Hefni et al. showed that the addition of dexmedetomidine to bupivacaine provides more effective postoperative analgesia than the addition of ketamine during pectoralis blocks in breast cancer surgery (22). However, in another study, adding 1 mg/kg of ketamine to 0.25% bupivacaine in an intercostal block in breast cancer surgery was shown to be useful, consistent with the results of the present study. In the ketamine and bupivacaine group, pain intensity and the need for analgesics were clinically lower than the bupivacaine group; however, their VAS score had no significant difference between the two groups (23).

In the current study, the hemodynamic variables in both groups were compared. Despite a clinically observed decrease in the MAP, HR, and RR in both groups, with a greater decrease in the dexmedetomidine group, no statistically significant difference was observed between the two groups. In line with the results of the present study, Dong et al. investigated the effect of dexmedetomidine-sufentanil versus sufentanil in a venous patient-controlled analgesia (PCA) pump after thoracotomy surgery. The dexmedetomidine-sufentanil group reported a lower pain score at rest and less coughing in the first 48 hours after the operation, with a three-fold decrease in the rate of nausea and vomiting, compared to the other group. However, there was no significant difference in terms of HR and blood pressure between the two groups (24). In a similar study, Ramsay et al. tested the effects of PCA pump dexmedetomidine versus placebo after surgery. The mean levels of HR and mean SBP were lower in the drug-receiving group, achieving a better sedation score (25). Future studies might consider investigating SAPB in other types of thoracic surgery. However, it is recommended to explore different doses of ketamine and dexmedetomidine to determine the appropriate and safe dosage associated with excellent improvement in analgesia efficacy.

### 4.1. Conclusions

In the present study, dexmedetomidine and ketamine were used as adjuvants to ropivacaine for SAPB in patients undergoing elective thoracotomy. They both reduced the pain intensity of the patients after thoracotomy; however, this pain reduction was more intense and effective in the group receiving ketamine.

## Clinical Trial Registration Code:

IRCT20190417043295N3.
